# Breastfeeding attributable fraction of triple negative breast cancer in the US

**DOI:** 10.1038/s41523-025-00755-6

**Published:** 2025-05-07

**Authors:** Rachel Jaber Chehayeb, Nicole Odzer, Roberta A. Albany, Leah Ferrucci, Daniel Sarpong, Rafael Perez-Escamilla, Jessica B. Lewis, Amanda I. Phipps, Allison Meisner, Lajos Pusztai

**Affiliations:** 1 Yale University School of Medicine, New Haven, CT USA; 2 Cancer-in-the-Know, Mt Penn, PA USA; 3 SWOG Clinical Trial Network, Seattle, WA USA; 4 Yale School of Public Health, New Haven, CT USA; 5 Department of Internal Medicine, Yale School of Medicine, New Haven, CT USA; 6 Department of Epidemiology, University of Washington, Seattle, WA USA; 7 Public Health Sciences Division, Fred Hutchinson Cancer Center, Seattle, WA USA; 8 Yale Cancer Center, New Haven, CT USA

**Keywords:** Risk factors, Cancer prevention, Breast cancer

## Abstract

Rates of triple negative breast cancer (TNBC) are higher in Black women than in non-Hispanic White women. Breastfeeding duration and younger age at first birth are known risk factors for TNBC and vary by race. To quantify the contribution of these risk factors to disparities in TNBC, we calculated the population-attributable fraction (PAF). A PubMed search was performed to identify relevant studies and pooled odds ratios for breastfeeding for < 6 months and age at first birth < 25 years were calculated. PAF was calculated using the Levin formula. PAF of breastfeeding for < 6 months was 12% (95% confidence interval (CI) 5–20%) among White women and 15% (95%CI 3–26%) among Black women. We estimate that up to 15% of annual new TNBC in Black women and 12% in White women might be avoided by supporting breastfeeding. Policies supporting breastfeeding could hence reduce TNBC incidence and lessen racial disparities.

## Introduction

Triple negative breast cancer (TNBC) is defined by the lack of estrogen (ER) or progesterone receptor (PR) expression and no amplification/overexpression of the human epidermal growth factor receptor 2 (HER2) gene. It is more commonly diagnosed in pre-menopausal women, and a minority of TNBC is associated with germline BRCA1/2 mutations^1^. Age-adjusted incidence rates of TNBC are higher in Black than in non-Hispanic White women. TNBC accounts for a greater proportion of breast cancers in Black vs. White women (19–28% vs. 9–14%)^2–5^. The cause(s) of this difference in incidence rate are unknown. Genetic predisposition has been proposed based on associations with ancestry-specific germline variants^6^. TNBC is more frequent in women with West African compared to East African ancestry and is associated with a higher frequency of Duffy blood group (Atypical Chemokine Receptor 1 [DARC/ACKR1]) null genotype (rs2814778) that provides protection against malaria but also disrupts cytokine networks relevant for cancer^7,8^.

Reproductive risk factors for TNBC development have also been identified. The lack of, or short duration of, breastfeeding and early pregnancy has been linked to increased TNBC risk in case-control and cohort studies for both White and Black women. Breastfeeding has also been shown to reduce the risk of developing luminal (ER-positive) disease^9^. Odds ratios (OR) for never breastfeeding compared to breastfeeding for > 6 months range from 1.37 (95% confidence interval [95%CI]:1.0–1.89) to 2.0 (95%CI: 1.11–3.33) for TNBC^10–13^. Many studies also identified multiple pregnancies and younger age at first birth as risk factors for TNBC^12,14–16^. There are differences in the incidence of these risk factors by self-reported race in the USA. In national surveys, 74% of Black women report having ever breastfed and 44% continued to breastfeed for 6 months, compared to 85% and 60%, respectively, for White women^17^. According to the most recent National Survey of Family Growth (2015–2019), 77% of Black women are younger than 25 years of age at first birth compared to 51% of White women^18^.

To what extent these differences in the prevalence of reproductive risk factors contribute to differences in TNBC incidence between White and Black women is unknown. To address this important question, we retrieved studies on breastfeeding and risk of TNBC in the US and, using pooled odds ratios as an estimate of relative risk, calculated the population attributable fraction (PAF). PAF quantifies the proportion of disease that is due to a particular risk factor exposure in a given population^19,20^. We also calculated the PAF of age at first birth < 25 years and the combined impact of short or no breastfeeding and early pregnancy. Using this metric, we estimated the number of new TNBC cases that could be attributed to potentially modifiable reproductive risk factors in White and Black women, respectively, in the US.

## Results

### Breastfeeding pooled odds ratio and PAF

**Fig. 1:**
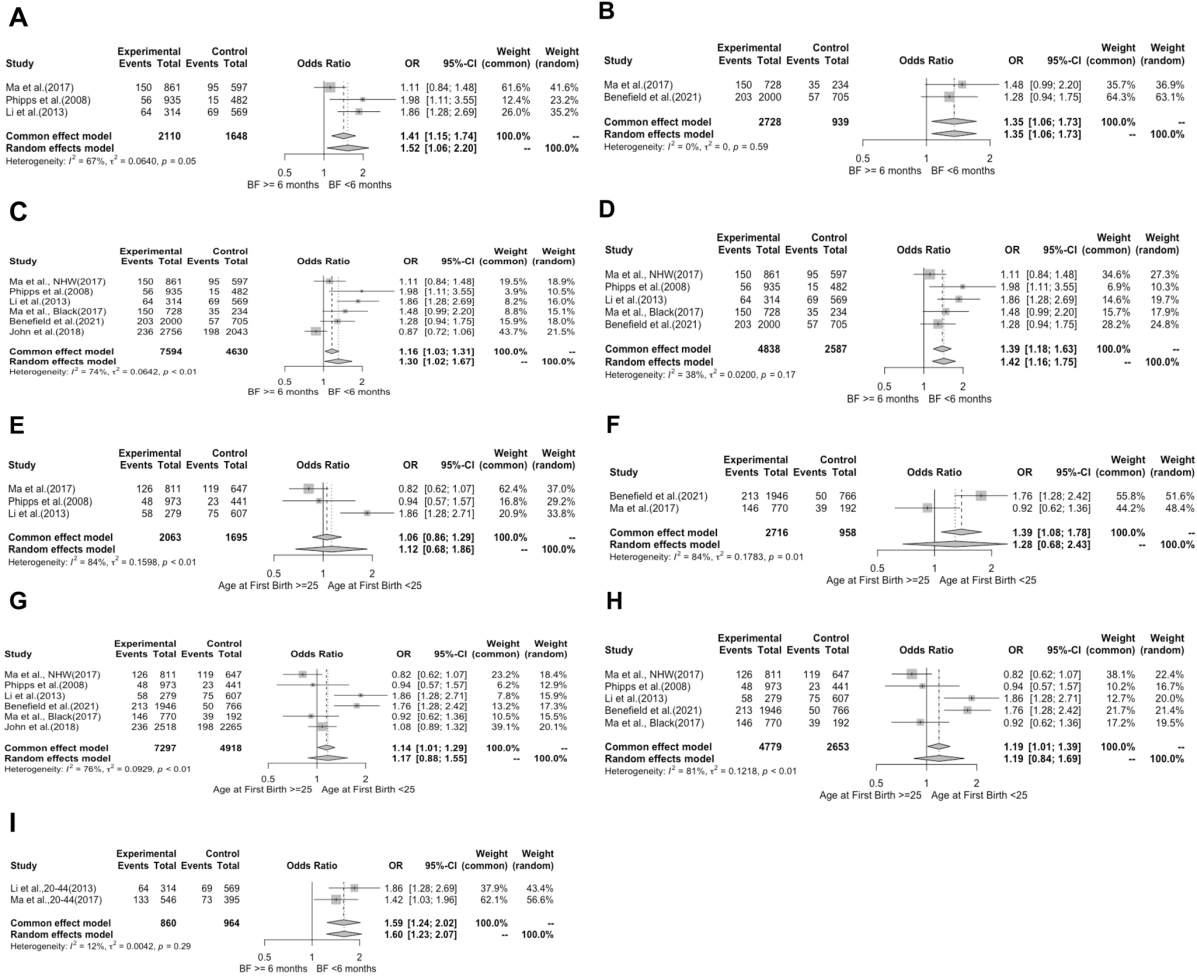
Forest plots showing pooled unadjusted OR for both White and Black women across reproductive risk factors. **A** Pooled OR for lack of breastfeeding for 6 months among White women. **B** Pooled OR for lack of breastfeeding for 6 months among Black women. **C**. Pooled OR for breastfeeding overall **D**. Pooled OR for breastfeeding overall excluding John et al. **E** Pooled OR for age at first birth White women. **F**. Pooled OR for age at first birth among Black women. **G** Pooled OR for age at first birth overall. **H** Pooled OR for age at first birth overall, excluding John et al. **I**. Pooled OR for lack of breastfeeding for 6 months among women aged 20–44.

Breastfeeding <6 months was associated with higher odds of TNBC in both White and Black women. The pOR among White women was 1.41 (95%CI: 1.15–1.74) with moderate heterogeneity ***I***^***2***^ = 67% (Table 1). Among Black women, the pOR was 1.35 (95%CI: 1.06–1.73) with minimal heterogeneity (***I***^***2***^ = 0). The overall pOR for TNBC from breastfeeding < 6 months was 1.16 (95%CI: 1.03–1.31), ***I***^***2***^ = 74%, and if John et al. were excluded, it was 1.39 (95%CI: 1.18-1.63), ***I***^***2***^ = *38****%***. For women aged 20-44 across all races, the pOR was 1.59 (95%CI: 1.24-2.02), ***I***^***2***^ = 12%. The confidence intervals for the pORs across all subgroups were overlapping (Fig. 1).

**Table 1:** Pooled Unadjusted OR for both White and Black Women Across Reproductive Risk Factors

	Risk Factors		
Subgroup		Breastfeeding <6 m	Age at first birth <25
White	**Pooled OR (95% CI)**	1.41 (1.15,1.74)	1.06 (0.86,1.29)
	***I***^***2***^***%***	67	84
	***P***^***a***^	0.05	<0.01
Black	**Pooled OR (95% CI)**	1.35 (1.06,1.73)	1.39 (1.08,1.78)
	***I***^***2***^***%***	0	84
	***P***^***a***^	0.59	0.01
Overall	**Pooled OR (95% CI)**	Common: 1.39 (1.18,1.63) Random: 1.42 (1.16,1.75)	Common: 1.19 (1.01,1.39) Random: 1.19(0.84,1.69)
	***I***^***2***^***%***	38	81
	***P***^***a***^	0.17	<0.01
Overall with et al.	**Pooled OR (95% CI)**	Common: 1.16 (1.03,1.31) Random: 1.30 (1.02,1.67)	Common: 1.14 (1.01,1.29) Random: 1.19 (0.88,1.55)
	***I***^***2***^***%***	74	76
	***P***^***a***^	<0.01	<0.01
Overall for women aged 20-44	**Pooled OR (95% CI)**	1.59 (1.24,2.02)	-
	***I***^***2***^***%***	12	-
	***P***^***a***^	0.29	-

^*a*^*P* for heterogeneity.

Using race-specific ORs, the PAF of breastfeeding <6 months was 12% (95%CI: 5–20%) among White women and 15% (95%CI 3–26%) among Black women. These race-specific values did not significantly differ from *PAF* using overall pOR across all studies except when including John et al. (Table 2). Among women aged 20-44, the PAF of breastfeeding <6 months was 18% (95%CI: 8-–28). Across all women, the PAF was 13% (95%CI 6-9) excluding John et al., and 6% (95%CI 0–0) when including the single outlier John et al.

**Table 2:** PAF Calculations for Both White and Black women Across Reproductive Risk Factors^a^

Risk Factor	White PAF (95% CI)	Black PAF (95% CI)	White using Pooled OR PAF (95% CI)	White using Pooled OR w/o John PAF (95% CI)	Black using Pooled OR PAF (95% CI)	Black using Pooled OR w/o John PAF (95% CI)	All Women without John PAF (95% CI)	All Women with John PAF (95% CI)	Women aged 20-44 PAF (95% CI)
Breastfeeding < 6 m vs >= 6 m	12 (5,20)	15 (3,26)	5 (0.3,9)	11 (6,17)	7 (0.5,13)	16 (8,23)	13 (6,19)	6 (0,1)	18 (8,28)
Age at first birth <25	2 (-6,11)	21 (5,35)	6 (0.4,11)	7 (0.4,14)	9 (1,16)	11 (1,21)	9 (1,17)	7 (1,13)	-
Combined PAF	12	26.7	8.6	13.9	12.4	20.5	16.9	10.1	-

^a^PAF was calculated only for significant OR (*p*-value < 0.05).

### Age at first birth, pooled odds ratio, and PAF

Having a child prior to age 25 was associated with a significantly higher risk of TNBC in Black women (pOR=1.39, 95%CI: 1.08–1.78), but not in White women (pOR=1.06, 95%CI 0.86-1.29), with significant heterogeneity in both groups (***I***^***2***^ = 84% for both). Overall, the pOR using the common-effects model was 1.19 (95%CI: 1.01–1.39), ***I***^***2***^ = 81% when we excluded John et al., and it was 1.14 (95%CI: 1.01–1.29), ***I***^***2***^ = 76% when including John et al. For all women, PAF for age at first birth <25 years was 7% (95% CI: 1–13), and 9% (95%CI :1–17) when excluding John et al. Overall, the PAF for age at first birth <25 years ranged from 2% (95%CI: -6–11) for White women to 21% (95%CI: 5–35) among Black women.

### Combined PAF calculations

Different approaches to estimating the polychoric correlation coefficient between age at first birth and breastfeeding as ordinal categories (never, 0-6, 6 + ; 0–6, 6–12, 12–18, 18 + ) across racial groups (all, Black-specific, White-specific) consistently yielded an estimated coefficient of ~0.2 which was used across combination PAF calculations (Supplemental Table 1). The combined PAF for breastfeeding <6 months and age at first birth <25 years ranged from 8.6% among White women to 26.7% for both Black women using race-specific pOR (Table 2). The combined PAF across all women was 16.9% when excluding John et al. and 10.1% when including John et al.

### Number of annual new TNBC attributable to lack of breastfeeding and early parity

Of all TNBC diagnosed in 2022, 4850 were attributable to breastfeeding for less than 6 months and/or age at first birth less than 25 years of age. In White women 2421 and in Black women 1533, annual TNBC cases can be attributed to no breastfeeding or breastfeeding for less than 6 months and/or age at first birth younger than 25. Furthermore, in White and Black women, 12% (*n* = 2421) and 15% (*n* = 861) of annual TNBC cases, respectively, could be specifically attributable to breastfeeding < 6 months.

## Discussion

To our knowledge, our paper is the first to estimate the population attributable fraction of TNBC due to no or short duration of breastfeeding and age at first birth younger than 25 years in Black and White women in the USA. Based on our calculations, of the 5742 annual cases of TNBC among Blacks in the US, approximately 15% (*n* = 861) could be attributable to breastfeeding < 6 months. Of the 20,181 TNBCs among White women, 12% (*n* = 2421) are attributable to breastfeeding <6 months. Promoting initiation of breastfeeding and supporting continued breastfeeding for the minimum recommended 6 months duration could reduce TNBC incidence in all women and could also reduce disparities in TNBC incidence among Black and White women^2–5^.

A few earlier studies reported PAF of reproductive risk factors for TNBC in the general population. The study by Millikan et al. calculated a joint PAF of 53% for never breastfeeding and for increased waist-to-hip ratio (defined as > 0.77) for TNBC in all women, and 68% and 57% for premenopausal and postmenopausal Black women, respectively^21^. Their reported OR for never vs ever breastfeeding overlaps with our pooled OR for breastfeeding <6 months but the risk factor prevalence estimates differ between our studies. Millikan et al assumed a 76% never breastfed prevalence in Black women, while we used a CDC survey result that showed never breastfed prevalence of 25.9%^17^. Several older studies also calculated the PAF of breastfeeding for all breast cancer subtypes combined and found a protective effect of both breastfeeding and younger age at first birth. Results of these studies primarily reflect the protective effect of pregnancies for estrogen receptor-positive breast cancer, which accounts for over 75% of all breast cancers^22–26^. It is important to note that age at first pregnancy has a different association for breast cancer risk by receptor subtype. In particular, initiation of childbearing at a younger age is protective for luminal (ER-positive) breast cancer subtypes, which account for the majority of breast cancers in both Black and White women^9^. Moreover, the decision on when to have a child is a deeply personal one influenced by a variety of factors. Hence, while we do report PAF for age at first birth in the results given its epidemiological significance as a risk factor for TNBC, we believe that the primary value of our study is its potential public health message regarding promoting breastfeeding as a mechanism for reducing TNBC risk in Black women as well as White women.

Factors associated with successful breastfeeding ≥ 6 months include knowledge of benefit from breastfeeding for the infant and mother, presence of social support, access to communal networks (e.g., breastfeeding counselors and support groups), presence of role models (i.e. older family members who breastfed), working hour flexibility and ability to pump^27,28^. Factors linked to lower breastfeeding rates include prejudiced public perceptions, difficulty with milk expression or pain, financial challenges limiting access to breast pumps, and policy barriers in the workplace, such as limited maternity leave, unsupportive workplace culture, and lack of lactation rooms and breaks during work to pump^28,29^. Despite these barriers, it is important to note that 44% of Black women and 60% of White women do breastfeed >6 months, and large within-group differences exist. Non-U.S.-born Black women in the U.S. have higher breastfeeding rates than both U.S.-born Black and White mothers, highlighting the role of cultural factors and U.S. historical legacies (e.g., wet nurses, medical racism) in shaping breastfeeding attitudes^30^. These results also indicate that breastfeeding is a modifiable risk factor for TNBC.

Access to health care provider support for initiating breastfeeding and navigating challenges of continued breastfeeding are important^28^. Hence, it is critical that birthing facilities have plans in place to support breastfeeding, especially among women of color and working parents^31,32^. A survey of 283 US hospital administrators between 2019 and 2020 showed that only half had a plan to facilitate breastfeeding, including providing affinity support groups or connecting pregnant women to lactation providers^31^. Engaging trusted community supports and engaging community partners to appropriately message breastfeeding promotion is also important. Further, policy interventions to increase universal workplace accommodations for breastmilk pumping (e.g., time, private space, storage) would be critical to enable continued breastfeeding by working women.

Our study has limitations. We calculated unadjusted odds ratio from raw case-control data, and these are likely an overestimate since we could not adjust for potential confounders. In the absence of comprehensive data sets that include both information on TNBC incidence and risk factor prevalence, we had to pool studies from different years and across different age groups. These limitations could explain why the unadjusted OR for breastfeeding for less than 6 months was <1 in John et al. The study by John et al. specifically pooled cancer registry data across four population-based studies spanning 1995 through 2009 requiring data harmonization with variable derivation, it had a higher representation of racial and ethnic minority populations (71% of TNBC cases and 81% of controls), and a higher representation of women born abroad (38% of women under 50 and 35% of women over 50). Given the data harmonization and baseline demographic differences, there could be hidden confounding factors that made this study an outlier. We used a fixed-effect model to calculate the pOR. A random effects model is better suited to account for heterogeneity across the studies, but we had too few studies to accurately estimate inter-study variance. Finally, in the absence of risk factor prevalence data specifically for women aged 20-44, we used overall risk factor prevalence to calculate PAF in that age group. Despite these limitations, this study provides the best estimate of breastfeeding and parity-related PAF for TNBC.

In conclusion, we provide evidence supporting the benefits of breastfeeding not only for children but also for their mothers. We estimate that up to 15% of annual new TNBC in Black women and 12% in White women might be avoided by better supporting breastfeeding. Policy changes aimed at supporting and more broadly enabling breastfeeding, addressing structural barriers, and promoting a culture shift could reduce overall incidence and racial disparities in TNBC incidence in the USA. Increasing awareness of the protective role of breastfeeding, improving workplace policies, and limiting the lobbying power of formula companies might increase breastfeeding rates and duration, leading to healthier infants and fewer breast cancers.

## Methods

### Selection of relevant studies

**Fig. 2:**
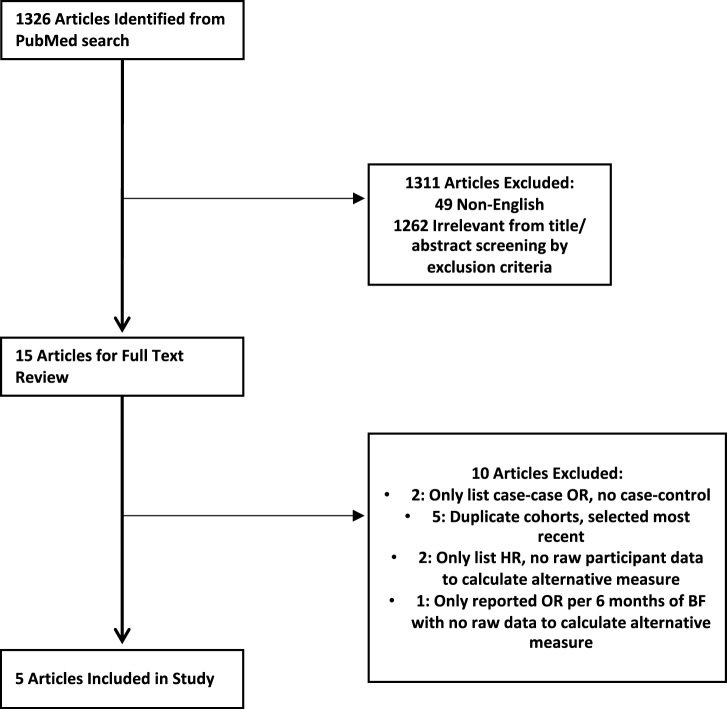
Flowchart showing search and study selection process.

A PubMed search was performed to identify articles from US-based case-control and cohort studies on the association between breastfeeding and the risk of TNBC. We used the search terms: breast cancer and breastfeeding; TNBC and breast feeding; breast cancer and lactation; breast cancer and breastfeeding; TNBC and breastfeeding; TNBC and lactation. We set 2008 as our publication year cut-off because HER2 testing in breast cancer was not widespread before that date and therefore TNBC status could not be confidently assigned^33^. The PubMed search was performed on September 4, 2023 and yielded 1326 results (Fig. 2). Titles and abstracts were screened by R.J.C and N.O to identify papers for full-text review. To be included, a study had to be a prospective cohort or case-control study set in the US, examine breastfeeding as an exposure in relation to TNBC or basal-like cancer specifically, and report odds ratio (OR), relative risk (RR), or hazards ratio (HR)^34^. Of the 15 articles that underwent full text review, 10 were excluded (Supplemental Table 2)^35^. At the end, data from 5 case-control studies (Supplemental Table 3) were used to compute PARs^10–12,15,36^. Since younger age at first birth was also an independently significant risk factor in two of the five studies^33,35^, we also calculated pooled OR for age at first birth <25 years compared to >25 as a secondary analysis. The following information was extracted from each of the studies: year, study design, cohort, risk factors examined, risk factors adjusted or matched for, maximally adjusted OR, and numbers of cases and controls.

### Calculation of pooled odds ratios

Pooled ORs and 95%CI were calculated, and forest plots were created to show the association between breastfeeding (nor or < 6 versus ≥ 6 months duration) and age at first birth (<25 versus ≥25 years of age). In deciding pooled ORs cut-offs, we aimed to homogenize across studies using cut-offs available in all studies, maximize sample sizes, and match study case control data to risk factor prevalence data from national surveys. Using adjusted maximal OR from the cohort studies was not feasible since each study used somewhat different breastfeeding cut-offs and calculated adjusted OR only between their chosen cutoff and never breastfeeding^11^. Six months of breastfeeding was chosen because it was the only duration cut-off, apart from ever or never breastfeeding, that was available in all included studies and was also available in the population prevalence surveys. Also, the American Academy of Pediatrics and the World Health Organization recommend exclusive breastfeeding for at least six months followed by continued breastfeeding for up to 2 years^37,38^.

To assess heterogeneity among studies for OR effect size and to generate a pooled OR (pOR) for all combined studies, we used the R package *meta*, which includes functions to fit both fixed and random effect models using the Mantel-Haenszel and inverse variance method approaches respectively as well as the Paule-Mandel estimator for ${\tau }^{2}$, a measure of interstudy variance^39–41^. We used the *I*^*2*^ statistic, which indicates the degree of variability in the observed effect that is explained by heterogeneity in association, and the p-value of the *Q* statistic to evaluate heterogeneity among studies, where Q is the chi-squared statistic and the p-value is generated by the Chi-squared test^35,42^. In this context, p < 0.1 is considered significant given the lack of power of the heterogeneity test^43^. Higher *I*^*2*^ values indicate greater heterogeneity, with a value of 25%, 50%, or 75% indicating low, moderate, and 75% high heterogeneity, respectively^35,42^.

For both White and Black subgroups, fixed ORs (common effect model) were calculated across chosen cut-offs (Supplemental Table 4). We also used all case-control data without stratification by race to calculate an overall pOR. For the pOR calculation, we included data from John et al., which was excluded from the Black- and White-women specific OR calculation as this study was not race-stratified^15^. Since John et al. was the only study in which the unadjusted OR for breastfeeding for less than 6 months was <1, we also reported pOR without John et al. to investigate the sensitivity of the pORs to the John et al. study. Given that TNBC disproportionately impacts younger women compared to other breast cancers, we calculated a pOR for breastfeeding <6 months for women across racial groups among younger women (aged 20-44)^12,36^.

All statistical analyses were conducted with R 4.1.3 statistical software and Python 3.12.

### Population prevalence data sources

Population-level breastfeeding estimates were obtained from the CDC’s National Immunization Survey^17^. Population-level parity and age at first birth data were extracted from a report on the National Survey of Family Growth (https://www.cdc.gov/nchs/nsfg/index.htm) that includes information on self-reported race^18^.

### PAF calculation

To calculate PAF, we used the Levin formula^44,45^:

1 $${PAF}=\frac{{P}_{{RF}}\times ({RR}-1)}{1+{P}_{{RF}}\times ({RR}-1)}$$

where P_RF_ is the population prevalence (%) of a risk factor. We used the pOR to approximate the RR given that TNBC is rare in the overall population^46^. We calculated the PAF for both the pooled unadjusted OR as well as for each individual study to show the variation in calculations depending on information source and to capture the range of possible PAFs. For race-specific PAF calculations, we calculated three different PAF measurements: (1) using race-specific OR, (2) using overall OR, and (3) using overall OR excluding John et al. We also calculated PAF for women aged 20-44 using specific pOR and overall population prevalence data (Supplemental Table 5).

### Combined PAF calculation

To calculate a combined PAF that accounts for the contribution of multiple risk factors, we adapted the method by Niedhammer and Chastang^47^:

2 $${\rm{Combined\; PAF}}=1-(1-v(i){\rm{PAF}}1)\times (1-v(i){\rm{PAF}}2))$$

where *v(i)=1-* r, where r is the polychoric correlation coefficient between breastfeeding duration and age at first birth as ordinal variables (Supplemental Table 1). Polychoric correlations between age at first birth and breastfeeding as ordinal variables were calculated from the publicly available 2021 CDC National Immunization Survey data (https://www.cdc.gov/vaccines/imz-managers/nis/index.html) using the Python package GIRTH (https://eribean.github.io/girth/#td-block-1). Given that breastfeeding duration and age at first birth are only applicable to parous women, we calculated the corresponding P_RF_ by multiplying the prevalence of parous women with the prevalence of breastfeeding duration or age at first birth, respectively (Supplemental Table 6).

### Estimating the annual number of TNBC cases attributable to no/short duration of breastfeeding and early parity

According to the Breast Cancer Statistics by the American Cancer Society, the total number of new invasive breast cancers diagnosed in 2022 was 287,850^2^. We used the reported racial breakdown of breast cancers (77.9% in White women and 10.5% in Black women between 1998–2018 to calculate the number of new invasive breast cancers per racial group^2,3^. We used the breast cancer subtype breakdown (10% TNBC across all races, 19% among Black women, 9% among White women) to calculate the number of annual TNBC per racial group^2^. We used the estimated number of new invasive cases of breast cancer in women less than 40 years of age (*N* = 10,850) to calculate the annual incidence of TNBC in women under 40 years of age^2^. We multiplied the annual number of newly diagnosed TNBC by self-reported race-specific PAF to estimate the number of new TNBC attributable to short breastfeeding and early parity each year.

## Supplementary information

Supplementary Material

## Data Availability

Data for each included study is available in the Supplementary Online Content.

## Code availability

The code for the statistical analysis performed in R, using the R package *meta*, and Python, using the package GIRTH, can be shared with interested readers upon request via email to the corresponding author.
