# Supplementary material for: Breastfeeding attributable fraction of triple negative breast cancer in the US
Source: NPJ Breast Cancer. 2025 May 7;11:40. doi: 10.1038/s41523-025-00755-6 (PMC12055980; doi:10.1038/s41523-025-00755-6)
Supplement: Supplementary file 1 — Supplementary Material [file 41523_2025_755_MOESM1_ESM.docx]

**Supplementary Material**

**Supplemental Table** **1**: Various Polychoric Correlations Calculation Across Different Breast-Feeding Cut-offs and Racial Groups

**Supplemental Table 2**: List of Studies Included in Full-Text Review and Reasons for Exclusion

**Supplemental Table 3:** Studies Included in Meta-Analysis

**Supplemental Table 4**: Breastfeeding and Age at First Birth Case and Control Data Across Studies

**Supplemental Table** **5**: Prevalence Rates of Risk Factors from Population-Based Surveys

**Supplemental Table 6**: Calculated PAF for Breast Feeding for Less than 6 months and Age at First Birth Less than 25 Years by Study and Overall.

**Supplemental Table 1: Various Polychoric Correlations Calculation Across Different Breast Feeding Cut-offs and Racial Groups**

| Race Stratification | Age at first birth  ordinal variable cut-offs | Breast feeding ordinal variable cut-offs | R polychoric correlation between age and duration |
| --- | --- | --- | --- |
| All (Black and White) | 0= 20-30, 1=30+ | 0=0-6 months  1=6-12 months  2=12-18 months  3=18 plus months | 0.19 |
| All (Black and White) | 0= 20-30, 1=30+ | 0= Never  1=0-6 months  2=6-12 months  3=12-18 months  4=18 plus months | 0.20 |
| All (Black and White) | 0= 20-30, 1=30+ | 0= Never  1=0-6 months  2=6+ months | 0.21 |
| White | 0= 20-30, 1=30+ | 0=0-6 months  1=6-12 months  2=12-18 months  3=18 plus months | 0.18 |
| Black | 0= 20-30, 1=30+ | 0=0-6 months  1=6-12 months  2=12-18 months  3=18 plus months | 0.21 |

**Supplemental Table 2: List of Studies Included in Full-Text Review and Reasons for Exclusion**

| Author, Year | Name | Included | If excluded, reason? |
| --- | --- | --- | --- |
| Ma et al.,2017[1] | Reproductive factors and the risk of triple-negative breast cancer in white women and African-American women: a pooled analysis | Yes |  |
| Chen et al., 2016 [2] | Reproductive Factors and Risk of Luminal, HER2-Overexpressing, and Triple-Negative Breast Cancer Among Multiethnic Women | No | includes only case-case OR (TNBC to non-TNBC, no comparison to controls) |
| Shinde et al., 2010 [3] | Higher parity and shorter breastfeeding duration: association with triple-negative phenotype of breast cancer | No | includes only case-case OR (TNBC to non-TNBC, no comparison to controls) |
| Palmer et al., 2014[4] | Parity, lactation, and breast cancer subtypes in African American women: results from the AMBER Consortium | No | included more recent 2021 study on AMBER cohort [5] that has better tumor molecular subtyping and a greater N (691 basal like breast cancers vs 567 TNBC cases) |
| Fortner et al., 2019[6] | Parity, breastfeeding, and breast cancer risk by hormone receptor status and molecular phenotype: results from the Nurses' Health Studies | No | only reports HR with no raw data available |
| Gaudet et al., 2011 [7] | Risk factors by molecular subtypes of breast cancer across a population-based study of women 56 years or younger | No | reports OR of breast feeding per 6 months with no raw case-control data |
| Li et al., 2013 [8] | Reproductive factors and risk of estrogen receptor positive, triple-negative, and HER2-neu overexpressing breast cancer among women 20-44 years of age | Yes |  |
| John et al., 2018[9] | Reproductive history, breast-feeding and risk of triple negative breast cancer: The Breast Cancer Etiology in Minorities (BEM) study | Yes (only in overall, does not stratify by race) |  |
| Phipps et al., 2008 [10] | Reproductive and hormonal risk factors for postmenopausal luminal, HER-2-overexpressing, and triple-negative breast cancer | Yes |  |
| Ambrosone et al., 2014 [11] | Parity and breastfeeding among African-American women: differential effects on breast cancer risk by estrogen receptor status in the Women's Circle of Health Study | No | Women's Circle of Health study data is included in subsequent AMBER study which we include[5] |
| Millikan et al., 2008 [12] | Epidemiology of basal-like breast cancer | No | reported data is part of subsequent AMBER study which we include[5] |
| Ma et al., 2010 [13] | Use of four biomarkers to evaluate the risk of breast cancer subtypes in the women's contraceptive and reproductive experiences study | No | uses the CARE study that is then pooled with subsequent studies in included 2017 paper by Ma et al. [1] |
| Chollet-Hinton et al., 2017 [14] | Biology and Etiology of Young-Onset Breast Cancers among Premenopausal African American Women: Results from the AMBER Consortium | No | included AMBER study, from 2021[5] |
| Sisti et al., 2015 [15] | Reproductive risk factors in relation to molecular subtypes of breast cancer: Results from the nurses' health studies | No | only reports HR, no raw data available |
| Benefield et al., 2021 [5] | Epidemiology of Basal-like and Luminal Breast Cancers among Black Women in the AMBER Consortium | Yes |  |

**Supplemental Table 3: Studies Included in Meta-Analysis**

| Author | Year | Study Design | Cohort | Racial group pre-dominance | TNBC Cases (n); age (years) | Risk Factors examined | Adjusted or matched for |
| --- | --- | --- | --- | --- | --- | --- | --- |
| Ma et al. [1] | 2017 | 3 case-control studies | Women’s Contraceptive and reproductive experiences study  Women’s breast carcinoma in site study  Women’s learning the influence of family and environment | Gives separate White and Black ORs | 554; 20-64 | Age at menarche. Number of completed pregnancies. Among parous women only: age at first completed pregnancy and duration of breast feeding (never, ever, <6,6-11, >12). | Adjusted for study source, study site, education level, race, age, family history, variable combining menopausal status and hormone therapy use, alcohol intake, smoking status, and OCP use. |
| Benefield et al. [5] | 2021 | case-control study | AMBER: Black women’s health study  Multiethnic cohort study  Carolina breast cancer study  Women’s circle of health study | Black only | 295 (basal, 691 overall but only 295 used in regression model); unspecified | Family history, age at menarche, age at first full-term birth, parity and breastfeeding, lifetime breastfeeding duration, oral contraceptive use, body mass index, waist to hip ratio. | Adjusted for age, family history, parity, breastfeeding duration, and study site. |
| Phipps et al. [10] | 2008 | case-control study | 2 pooled population based studies cases for both studies  were identified through the Cancer Surveillance System (CSS) of western Washington State. | White (90%) | 78; 55+ | age at menarche, parity, # of births,  age at first birth, breast feeding history,  type of menopause, age at menopause, hormone therapy use. | Adjusted for age and diagnosis/ reference year. Among parous women, number of live births, age at first live birth, and breast feeding are co-adjusted for each other. |
| Li et al.[8] | 2013 | Case-control study | Population-based case–control study. Cases were women with TNBC 20–44 years diagnosed between 2004-2010 in the Seattle–Puget Sound metropolitan | White(80%) | 184; 20-44 | age at menarche, parity, # of births, age at first birth, breast feeding history, type of menopause, age at menopause, hormone therapy use | Adjusted for age and diagnosis/ reference year. Among parous women, number of live births, age at first live birth and breast feeding are co-adjusted. |
| John et al. [9] | 2018 | Case-control study | Breast cancer etiology in minorities study  San Fransisco Bay Area Breast Cancer Study  Northern California site of Breast Cancer Family Registry (NC-BCFR)  Los Angeles Country Asian American Breast Cancer Study (AABCS)  4 Corners Breast Cancer Study (4-CBCS) | no single racial group predominance | 558; 18-79 | Age at first FTP; Parity by history of breast feeding; Parity (# of FTP); Breast feeding history; Duration of breast feeding; Parity by age at first FTP | Adjusted for base covariates, age, study, time period,education, family history, height, OCP use, menopausal status/hormone therapy use. |

**Supplemental Table 4: Breastfeeding and Age at First Birth Case and Control Data Across Studies.**

BF= breast-feeding, C= case, Ctl= control, AFB= age at first birth

| Author | Year | Never  or <6m BF  C | Never  or <6m  BF Ctl | 6+ BF C | 6+ BF Ctl | AFB <25 C | AFB <25 Ctl | AFB 25+ C | AFB 25+ Ctl |
| --- | --- | --- | --- | --- | --- | --- | --- | --- | --- |
| Ma et al., White | 2017 | 150 | 711 | 95 | 502 | 126 | 685 | 119 | 528 |
| Phipps et al. | 2008 | 56 | 879 | 15 | 467 | 48 | 925 | 23 | 418 |
| Li et al. | 2013 | 64 | 250 | 69 | 500 | 58 | 221 | 75 | 532 |
| Ma et al., Black | 2017 | 150 | 578 | 35 | 199 | 213 | 1733 | 50 | 716 |
| Benefield et al. | 2021 | 203 | 1797 | 57 | 648 | 146 | 624 | 39 | 153 |
| John et al. | 2018 | 236 | 2520 | 198 | 1845 | 236 | 2282 | 198 | 2067 |
| Li et al., 20-44 | 2013 | 58 | 221 | 75 | 532 | 58 | 221 | 75 | 532 |
| Ma et al, 20-44 | 2017 | 114 | 377 | 92 | 358 | 114 | 377 | 92 | 358 |

**Supplemental Table 5: Prevalence Rates of Risk Factors from Population-Based Surveys**

|  | **White Women** | **Black Women** | **All Women** |
| --- | --- | --- | --- |
| **Lack of breast feeding at 6 months (never or breast feeding for <6 months)[16]** | 40.1± 1.7% | 56 ± 3.7% | 44.2 ± 1.3 |
| **% of women parous by age 49 [17]** | 81.8% | 86.7% | 84.3 % |
| **% of women with age at first birth <25 [17]** | 50.9 ± 3% | 77.1±4.16% | 62.2% |

**Supplemental Table 6: Calculated PAR for Breast Feeding for Less than 6 months and Age at First Birth Less than 25 Years by Study and Overall.**

BF= breast feeding, AFB= age at first birth, PAR= population attributable risk, CI= confidence interval.

| Author | BF PAR % (95% CI) | AFB PAR % (95% CI) | Combined PAR% (95% CI) |
| --- | --- | --- | --- |
| White Using Pooled White OR | 12(5,20) | 2(-6,11) | 12 |
| Black using Pooled Black OR | 15(3,26) | 21(5,34) | 26.7 |
| Overall without John et al. | 13(6,19) | 9(1,17) | 16.9 |
| Overall | 6(0,1) | 7(1,13) | 10.1 |
| Black using Pooled Overall OR without John et al. | 16(8,23) | 11(1,21) | 20.5 |
| Black using Pooled Overall OR | 7(0.5,13) | 9(1,16) | 12.4 |
| White Using Pooled Overall OR without John et al. | 11(6,17) | 7(0.4,14) | 13.9 |
| White Using Pooled Overall OR | 5(0.3,9) | 6(0.4,11) | 8.6 |
| Overall for Women Aged 20-44 (Ma et al. and Li et al.) | 18(8,28) |  |  |

**Supplementary References**

1. Ma, H., et al., *Reproductive factors and the risk of triple-negative breast cancer in white women and African-American women: a pooled analysis.* Breast Cancer Res, 2017. **19**(1): p. 6.

2. Chen, L., et al., *Reproductive Factors and Risk of Luminal, HER2-Overexpressing, and Triple-Negative Breast Cancer Among Multiethnic Women.* Cancer Epidemiol Biomarkers Prev, 2016. **25**(9): p. 1297-304.

3. Shinde, S.S., et al., *Higher parity and shorter breastfeeding duration: association with triple-negative phenotype of breast cancer.* Cancer, 2010. **116**(21): p. 4933-43.

4. Palmer, J.R., et al., *Parity, lactation, and breast cancer subtypes in African American women: results from the AMBER Consortium.* J Natl Cancer Inst, 2014. **106**(10).

5. Benefield, H.C., et al., *Epidemiology of Basal-like and Luminal Breast Cancers among Black Women in the AMBER Consortium.* Cancer Epidemiol Biomarkers Prev, 2021. **30**(1): p. 71-79.

6. Fortner, R.T., et al., *Parity, breastfeeding, and breast cancer risk by hormone receptor status and molecular phenotype: results from the Nurses' Health Studies.* Breast Cancer Res, 2019. **21**(1): p. 40.

7. Gaudet, M.M., et al., *Risk factors by molecular subtypes of breast cancer across a population-based study of women 56 years or younger.* Breast Cancer Res Treat, 2011. **130**(2): p. 587-97.

8. Li, C.I., et al., *Reproductive factors and risk of estrogen receptor positive, triple-negative, and HER2-neu overexpressing breast cancer among women 20-44 years of age.* Breast Cancer Res Treat, 2013. **137**(2): p. 579-87.

9. John, E.M., et al., *Reproductive history, breast-feeding and risk of triple negative breast cancer: The Breast Cancer Etiology in Minorities (BEM) study.* Int J Cancer, 2018. **142**(11): p. 2273-2285.

10. Phipps, A.I., et al., *Reproductive and hormonal risk factors for postmenopausal luminal, HER-2-overexpressing, and triple-negative breast cancer.* Cancer, 2008. **113**(7): p. 1521-6.

11. Ambrosone, C.B., et al., *Parity and breastfeeding among African-American women: differential effects on breast cancer risk by estrogen receptor status in the Women's Circle of Health Study.* Cancer Causes Control, 2014. **25**(2): p. 259-65.

12. Millikan, R.C., et al., *Epidemiology of basal-like breast cancer.* Breast Cancer Res Treat, 2008. **109**(1): p. 123-39.

13. Ma, H., et al., *Use of four biomarkers to evaluate the risk of breast cancer subtypes in the women's contraceptive and reproductive experiences study.* Cancer Res, 2010. **70**(2): p. 575-87.

14. Chollet-Hinton, L., et al., *Biology and Etiology of Young-Onset Breast Cancers among Premenopausal African American Women: Results from the AMBER Consortium.* Cancer Epidemiol Biomarkers Prev, 2017. **26**(12): p. 1722-1729.

15. Sisti, J.S., et al., *Reproductive risk factors in relation to molecular subtypes of breast cancer: Results from the nurses' health studies.* Int J Cancer, 2016. **138**(10): p. 2346-56.

16. Raju, T.N.K., *Achieving healthy people 2030 breastfeeding targets in the United States: challenges and opportunities.* J Perinatol, 2023. **43**(1): p. 74-80.

17. Martinez, G.M. and K. Daniels, *Fertility of Men and Women Aged 15-49 in the United States: National Survey of Family Growth,2015-2019.* Natl Health Stat Report, 2023(179): p. 1-22.
